# The impact of glycosylated hemoglobin and 75-g oral glucose tolerance test glucose levels on birthweight percentile

**DOI:** 10.1590/1806-9282.20240466

**Published:** 2024-09-16

**Authors:** Yasemin Doğan, Ayşe Seda Daryal

**Affiliations:** 1 Kocaeli University, Department of Obstetrics and Gynecology, Perinatology Unit – Kocaeli, Turkey.; 2 Kocaeli University, Department of Obstetrics and Gynecology – Kocaeli, Turkey.

**Keywords:** Large for gestational age infant, Oral glucose tolerance test, Glycated hemoglobin A1c, Birthweight

## Abstract

**OBJECTIVE:**

Our research objective was to validate and contribute further evidence to the studies regarding large for gestational age and birthweight percentile by examining oral glucose tolerance test and glycosylated hemoglobin levels in both healthy women and those with gestational diabetes mellitus.

**METHODS:**

This retrospective cohort study was conducted at a tertiary care hospital involving 106 women who delivered at gestational week 36 or later between February 2022 and February 2023. Maternal, obstetric, and neonatal data were collected from the participant's medical records. Large for gestational age and non-large for gestational age groups were compared. Correlation analysis was used to determine associations among oral glucose tolerance test, glycosylated hemoglobin levels, and the birthweight percentile.

**RESULTS:**

Mothers of neonates in the large for gestational age category had higher body mass indexes before pregnancy (p=0.002) and delivery (p=0.003), as well as a higher incidence of gestational diabetes mellitus (p=0.027). Mothers of male large for gestational age infants had higher fasting plasma glucose and glycosylated hemoglobin levels compared to male non-large for gestational age infants (p=0.007 and p=0.004, respectively). There was a weak positive correlation between fasting plasma glucose levels and birthweight percentile in the overall group (r=0.342, p<0.006). Further analysis by gender showed a weak positive correlation between birthweight percentile and fasting plasma glucose and glycosylated hemoglobin values in male newborns (r=0.393, p=0.004 and r=0.373, p=0.006, respectively).

**CONCLUSION:**

Our study has established a clear association between the birthweight percentile in male infants and the levels of glycosylated hemoglobin and fasting plasma glucose measured during oral glucose tolerance test. It is imperative to devise potential strategies aimed at achieving optimal glycosylated hemoglobin and fasting plasma glucose parameters to effectively reduce the frequency of large for gestational age in male infants.

## INTRODUCTION

Birthweight is a crucial factor in predicting the survival of newborns and infants, as well as an important indicator of pregnancy outcomes^1^. Additionally, it has been observed that birthweight is associated with the future risk of cardio-metabolic diseases in the offspring. It was reported that 10.8% of newborns are large for gestational age (LGA), exhibiting a high birthweight percentile^2^.

Maternal glucose level is an important factor in prenatal health, as it plays a vital role in providing essential nutrients to both pregnant women and their fetuses^3^. GDM is a common metabolic disorder that refers to any degree of glucose intolerance occurring during pregnancy^4^. This condition has been linked to the passage of glucose through the placenta to the fetus, leading to increased fetal insulin production and subsequent excessive growth, resulting in LGA infants^5^. Although the diagnostic "gold-standard" for GDM is the oral glucose tolerance test (OGTT), glycosylated hemoglobin (HbA1c) is a commonly used measure to evaluate long-term glucose regulation^6^. Studies have established an association among GDM, high glucose levels, third-trimester HbA1c values, and LGA infants^7^.

While a significant portion of the research on prenatal glucose regulation has primarily concentrated on diabetic pregnancies, it is important to note that negative consequences have also been observed in pregnant individuals without diabetes^8^. However, there is a scarcity of studies, particularly focusing on pregnant women who do not have GDM. Our research objective is to validate and contribute further evidence to the studies regarding LGA and birthweight percentile by examining OGTT values and HbA1c levels in both healthy women and those with GDM.

## METHODS

This retrospective cohort study was conducted at Kocaeli University Hospital, involving 106 women who delivered at gestational week 36 or later between February 2022 and February 2023. Pregnant women with chronic hypertension, pre-gestational diabetes, preeclampsia, multiple pregnancies, or major fetal anomalies, as well as those with missing OGTT values, were excluded from the study. The study adhered to the ethical principles outlined in the 1964 Declaration of Helsinki and received approval from the Ethics Committee of Kocaeli University (KU GOKAEK 2023/17.16). Informed written consent was obtained from the patients.

Maternal, obstetric, and neonatal data, including age, pre-pregnancy height, weight and body mass indexes (BMI) before pregnancy and before delivery, gestational weight gain, OGTT values, HbA1c levels, gestational week of delivery, mode of delivery, neonatal weight, gender, APGAR scores, umbilical cord blood pH, and glucose levels, were collected from the participant's medical records. Gestational age was determined based on the patient's last menstrual period and confirmed by first-trimester crown-rump length.

Large for gestational age was defined as a birthweight that exceeds the 90th percentile. To screen for GDM, all patients underwent a 75-g OGTT between the 24th and 28th week of pregnancy. The criteria for diagnosing GDM were based on the International Association of Diabetes and Pregnancy Study Groups (IADPSG) guidelines, which consider a single high value during the 2-h 75-g OGTT as diagnostic for GDM^9^. The thresholds were fasting plasma glucose (FPG) level of 92 mg/dL, 1-h glucose level of 180 mg/dL, and 2-h glucose level of 153 mg/dL. Patients whose OGTT results fell within normal ranges were classified as having normal glucose tolerance. BMI was calculated by dividing the formula weight by the square of the height (kg/m^2^).

Glycosylated hemoglobin (%) levels were measured during the OGTT between 24 and 28 weeks of gestation. The calculation of the birthweight percentile was performed using the Fetal Medicine Foundation's neonatal population weight charts^10^.

### Statistical analysis

All statistical analyses were performed using IBM SPSS version 20.0 for Windows (IBM Corp., Armonk, NY, United States). Shapiro-Wilk and Kolmogorov-Smirnov tests were used to assess the normality assumption. Continuous variables were presented with mean±standard deviation or median (IQR: interquartile range). Categorical variables were summarized as counts and percentages. Independent-samples t-test or Mann-Whitney U test was used for comparison between groups. The association between two categorical variables was examined by the chi-square test. Spearman correlation analysis was used to determine the associations between continuous variables. A p-value <0.05 was considered statistically significant.

## RESULTS

This study included a total of 106 women. The mean maternal age was 30 ±5.6 years. The gestational age at the time of delivery varied between 36 and 40 weeks, with a mean of 38 weeks. The mean birthweight was 3400±458 g. There were 53 male and 53 female newborns. Maternal, obstetric, and neonatal characteristics of the study group are displayed in Table 1.

**Table 1:** Maternal, obstetric, and neonatal characteristics of the study population.

Maternal/obstetric variables		
Maternal age (years)		29 (26–34)
Pre-pregnancy weight (kg)		66.8±13.6
Pre-pregnancy BMI (kg/m^2^)		65 (58–73)
Gestational weight gain (kg)		12 (10–16)
Weight before delivery (kg)		77 (71–86)
BMI before delivery (kg/m^2^)		29.6 (26.7–33.7)
Parity		
	Nulliparous	37 (34.9%)
	Multiparous	69 (65.1%)
Mode of delivery		
	Vaginal delivery	30 (28.3%)
	Cesarean delivery	76 (71.7%)
	GDM	23 (21.6%)
	HbA1c (%)	5.2 (4.9–5.3)
	FPG (mg/dL)	82 (76–87)
	1-h OGTT (mg/dL)	132 (114–152)
	2-h OGTT (mg/dL)	109 (93–124)
Neonatal variables		
	GA at delivery (weeks)	38 (38–39)
	Birthwieght (g)	3400±458
	Birthweight percentile	63 (31–93)
	1st minute APGAR score	8 (7–8)
	5th minute APGAR score	9 (9–9)
	Umblical cord blood pH	7.33 (7.30–7.36)
	Umblical cord blood glucose (mg/dL)	75 (66–93)
Neonatal gender		
	Female	53 (50%)
	Male	53 (50%)

Data are shown as mean ± SD or median (25th–75th quartile) or number and percentage (%). BMI: body mass index; GDM: gestational diabetes mellitus; FPG: fasting plasma glucose; OGTT: oral glucose tolerance test; GA: gestational age.

Among 106 women, 21.6% (n=23) were diagnosed with GDM. LGA was present in 33% of the study group (n=35) while 67% (n=71) had non-LGA births. Table 2 displays the comparison of LGA and non-LGA groups for maternal, obstetric, and neonatal variables. There was a statistically significant difference in various factors such as pre-pregnancy BMI (p=0.002), BMI before delivery (p=0.003), GDM (p=0.027), and polyhydramnios (p=0.003). Additionally, the FPG values were significantly higher in the LGA group (p<0.001). LGA was more prevalent among male infants (p=0.013). One woman in the LGA group and four women in the non-LGA group used insulin (p=1). Only one woman in the LGA group and two women in the non-LGA group had unregulated glucose levels at the time of birth.

**Table 2:** Characteristics of the study population according to birthweight percentile categories.

			LGA	Non-LGA	p-value*
Maternal age (years)			30 (27–36)	29 (26–33)	0.405^a^
Pre-pregnancy weight (kg)			73.8±15.6	63.3±11.1	0.001^b^
Pre-pregnancy BMI (kg/m^2^)			28.3±6.1	24.6±4.3	0.002^b^
Gestational weight gain (kg)			12 (10–17)	12 (10–16)	0.637^a^
Weight before delivery (kg)			84 (75–97)	75 (67–82)	0.001^a^
BMI before delivery (kg/m^2^)			33.8 (27.7–37.3)	29.3 (26.7–31.6)	0.003^a^
Parity					
		Nulliparous	13 (37.1%)	30 (42.3%)	0.769^c^
		Multiparous	22 (62.9%)	41 (57.7%)	
Mode of delivery					
		Vaginal delivery	3 (8.6%)	27 (38.0%)	0.003^c^
		Cesarean delivery	32 (91.4%)	44 (62%)	
		GDM	12 (34.3%)	11 (15.4%)	0.027^c^
		NonGDM	23 (65.7)	60 (84.6%)	
		HbA1c (%)	5.2 (5.0–5.5)	5.1 (4.9–5.3)	0.102^a^
		FPG (mg/dL)	85 (82–91)	79 (74–86)	<0.001^a^
		1-h OGTT (mg/dL)	138 (118–164)	128 (111–145)	0.078^a^
		2-h OGTT (mg/dL)	109 (97–128)	110 (92–123)	0.450^a^
		GA at delivery (weeks)	37 (37–38)	38 (38–39)	0.007^a^
		Birthweight (g)	3877±237	3165±343	<0.001^b^
Amniotic fluid index					
		Polyhydramnios	8 (22.9%)	2 (2.9%)	0.003^c^
		Normal	27 (77.1%)	67 (97.1%)	
Umblical cord blood pH					0.125^a^
		Umblical cord blood glucose (mg/dL)	74.3±14.2	81.8±23	0.063^b^
Neonatal gender					
		Female	11 (31.4%)	42 (59.2%)	0.013^c^
		Male	24 (68.6%)	29 (40.8%)	

Data are shown as mean ± SD or median (25th–75th quartile) or number and percentage (%).

* p<0.05 indicates statistical significance.

a Independent-samples Mann-Whitney U test.

b Independent samples t-test.

c Chi square test. LGA: large for gestational age; BMI: body mass index; GDM: gestational diabetes mellitus; FPG: fasting plasma glucose; OGTT: oral glucose tolerance test; GA: gestational age.

Upon categorizing the groups by gender, it was observed that mothers of male LGA infants had higher levels of FPG and HbA1c compared to male non-LGA infants (p=0.007 and p=0.004, respectively) (Table 3).

**Table 3:** Comparison of glycosylated hemoglobin and oral glucose tolerance test values in large for gestational age and non-large for gestational age groups based on neonatal gender.

	Female		p-value*	Male		p-value*
	LGA	Non-LGA		LGA	Non-LGA	
HbA1c (%)	5.04±0.50	5.16±0.31	0.480^a^	5.35±0.44	5.02±0.32	0.004^a^
FPG (mg/dL)	85 (82–91)	78 (74–86)	0.079^b^	86 (80–90)	80 (73–84)	0.007^b^
1-h OGTT (mg/dL)	138 (122–155)	127 (105–144)	0.215^b^	138 (111–165)	131 (114–146)	0.288^b^
2-h OGTT (mg/dL)	108 (100–124)	109 (91–123)	0.775^b^	111 (91–133)	110 (92–121)	0.381^b^

Data are shown as mean ± SD or median (25th–75th quartile).

* p<0.05 indicates statistical significance.

a Independent-samples t-test.

b Mann-Whitney U test. LGA: large for gestational age; FPG: fasting plasma glucose; OGTT: oral glucose tolerance test.

According to correlation analysis, no significant correlations were found between birthweight percentile and the results of 1-h OGTT, 2-h OGTT, and HbA1c (p=0.195, p=0.546, and p=0.245 respectively), but FPG level showed a weak positive correlation (r=0.342 and p<0.006) in the overall group. Further analysis stratified by gender revealed that birthweight percentile exhibited a weak positive correlation with FPG and Hba1c values in male newborns (r=0.393, p=0.004 and r=0.373, p=0.006, respectively). However, the birthweight percentile of female infants was not associated with FPG or HbA1c values (p=0.159 and p=0.42, respectively).

## DISCUSSION

In our research, mothers of LGA infants tended to have a higher pre-pregnancy BMI and elevated FPG values. Our study found a correlation between the birthweight percentile and the neonatal gender, indicating a higher likelihood of classifying male newborns as LGA. Furthermore, we observed a positive correlation between HbA1c and FPG values and the birthweight percentile, particularly in male infants.

In our study, both maternal pre-pregnancy BMI and BMI before delivery were higher in the LGA group. Several research findings have indicated that a high pre-pregnancy BMI is the primary indicator for the delivery of a baby with fetal overgrowth. According to the study by Chen et al., the risk of macrosomia was significantly higher in women who were overweight or obese before pregnancy compared to women with a normal weight^11^. In another study, obese women were found to have a 2.27-fold higher likelihood of developing LGA compared to women who were not obese^12^. This observation was not limited to women with GDM but also included women with normal glucose tolerance. A large study from China underlined that male neonates, overweight, and obesity were linked with an increased risk of delivery higher than 4,000 g in nondiabetic women^13^.

The presence of GDM can have significant implications for both the mother and the fetus. The IADPSG criteria were formulated based on the findings of the hyperglycemia and adverse pregnancy outcomes (HAPO) study. The HAPO study revealed a consistent and progressive correlation between fasting and post-load maternal glucose levels and the occurrence of LGA infants, high adiposity, and elevated concentrations of cord-blood C-peptide^14^.

During pregnancy, blood glucose levels of 70 mg/dL or lower in the OGTT have been associated with lower birth weight, smaller head circumference, and shorter body length in infants compared to those born to mothers with normal blood glucose levels^15^. Individuals with a lower glucose challenge test (GCT) result during the second trimester had a higher incidence of SGA newborns compared to those with a normal result^16^. On the other hand, studies have underlined that the infants born to women with high FPG levels during OGTT exhibited a significantly greater average birthweight and birthweight percentile when compared with nonGDM women and a higher risk of LGA^17,18^. Zhao et al. found that as FPG levels increased by 1 mmol/L, there was a corresponding rise in the birth weight percentile^19^. They also observed a 0.70 times decrease in the risk of SGA, while LGA increased by 1.80 times. Glucose measurements exhibited a linear correlation with LGA, with the strongest link observed for FPG. In line with the aforementioned studies, we found that median FPG levels were higher in mothers of LGA fetuses. Further analysis showed that FPG levels were correlated with the birthweight percentile in our study. On the contrary, in a recent study, there was no significant correlation observed between FPG, plasma glucose 2 h after the OGTT, postprandial blood glucose values at 28 weeks of gestation, and birthweight^20^. According to Yang et al., fetal growth is mainly influenced by postload glucose levels rather than FPG values^21^. Nevertheless, the methodologies employed in the latter two studies differ from our own.

In a recent study, no significant relationship was found between the levels of maternal HbA1c before 20 weeks of gestation and the birthweight of the neonate in women without pre-existing diabetes^22^. Silke et al. observed a positive correlation between maternal HbA1c levels and neonatal birthweight^23^. However, this association was found to be significant only in pregnancies with male fetuses. Similarly, our research revealed a positive correlation between maternal HbA1c, FPG, and birthweight percentile, but exclusively among male neonates. It is important to note that birthweight percentile rather than birthweight was evaluated in our study. A prior investigation presented proof that the glucose metabolism of mothers could potentially be influenced by the gender of the fetus, even in pregnant women with normal glucose tolerance^24^. FPG was found to be higher in mothers of male infants. The presence of a male fetus was linked to diminished β-cell function, which refers to the reduced ability of pancreatic cells involved in counteracting insulin resistance^25^.

The study's strengths lie in the balanced representation of both genders, which enhances the statistical validity. Notably, our study encompassed a substantial number of healthy women, which has offered additional understanding and the benefit of placing various pathological glucose response patterns into context.

Acknowledging the study's limitations, reliance on self-reported pre-pregnancy height and weight may introduce recall bias. To minimize this, the participant's pre-pregnancy weights and heights were recorded at the onset of pregnancy. The study population consists primarily of individuals of Caucasian ethnicity, limiting the generalizability of our findings to other ethnic groups. The variable "birthweight" is influenced by numerous predictors and confounders, but our research is limited by a restricted set of predictor variables. Data on patient's dietary habits, physical activity, parental birthweight, and paternal body habitus are lacking, which could potentially impact the outcomes of the study.

## CONCLUSION

Our study has established a clear association between the birthweight percentile in male infants and the levels of HbA1c and FPG measured during OGTT. Pregnant women with abnormal glucose tolerance carrying a male fetus may require closer monitoring of fetal growth. It is imperative to devise potential strategies aimed at achieving optimal HbA1c and glucose parameters to effectively reduce the frequency of LGA in male infants, which should be considered in future research.

## ETHICAL APPROVAL

This study was carried out at Kocaeli University Hospital. All procedures performed in studies involving human participants were in accordance with the ethical standards of the institutional and/or national research committee and with the 1964 Helsinki Declaration and its later amendments or comparable ethical standards. This study received approval from the Ethics Committee of Kocaeli University (KU GOKAEK 2023/17.16). Informed written consent was obtained from the patients.

## Data Availability

The data supporting this study are available through the corresponding author upon reasonable request.
